# Debate: Young people are living in unprecedented times – too much chaos or too little resilience?: Are today's young people really less resilient or have we created systems that strip away the experiences resilience needs to grow?

**DOI:** 10.1111/camh.70103

**Published:** 2026-07-15

**Authors:** Lucy Bowes

**Affiliations:** ^1^ Department of Experimental Psychology Magdalen College, University of Oxford Oxford UK

**Keywords:** Resilience, child and adolescent, allostatic load, challenges, opportunities

## Abstract

Children and young people's mental health appears to be worsening, despite marked declines in many forms of severe adversity over recent decades. This article argues that young people's distress and withdrawal from society are understandable responses to an environment that is simultaneously over‐protective in some respects and chronically threatening in others. It calls for more systemic thinking in how we support children, expanding opportunities for face and overcome manageable challenges, while acknowledging the very real structural stressors shaping their lives.

Most mornings now, you only have to glance at the news on your phone to feel as if you have entered a dystopian nightmare: biodiversity is collapsing, World War Three is coming, and AI may threaten the very fabric of our society. The headlines capture a wider sense that the world our children are growing up in is becoming increasingly chaotic. Yet in many fundamental ways, our children's lives have never been better. Globally, they are much less likely to die before their fifth birthday than at any other point in human history. Records of child cruelty or neglect have fallen sharply over the past 150 years, even though child maltreatment remains a major public health concern. Access to schooling has expanded worldwide, child exploitation has declined and legal protections for children have been strengthened. At the same time, concern about young people's mental health has intensified. Rates of anxiety and depression continue to increase, and mental health difficulties are commonplace in our schools and universities. Are we failing to build resilience in this generation or are young people responding adaptively to a world we have created for them? In this essay, I argue that, while we have achieved major public health successes and reduced many forms of serious harms, chronic structural stressors have intensified. At the same time, we may have unintentionally reduced everyday opportunities to learn from manageable stress. Drawing on contemporary resilience research, I consider how we might apply the kind of careful cost–benefit thinking that is routine in medicine to decisions about children's exposure to risk, how modern forms of chronic stress are shaping children's lives, and what a systems‐level view of resilience implies for policy and practice.

Childhood has become safer in terms of extreme adversity. Official records in England and Wales indicate that violent child deaths have decreased by about 90%, and convictions for child cruelty or neglect have dropped by more than 80% since the late 1800s (Degli Esposti et al., 2019). Across recent decades, children in many high‐income countries are less likely than previous generations to experience early parental or sibling death, serious childhood illnesses, extreme poverty or physical and sexual abuse (Finkelhor, Shattuck, Turner, & Hamby, 2020; Ritchie, 2025). These are enormous public health successes that should not be minimized. Yet alongside this, I believe our young people are today facing different, more pervasive stressors. Many young people report feeling hopeless about their future and are anxious about the growing threats of climate change and geopolitical instability. They face very real pressures of economic uncertainty and unemployment. Families are facing financial strain, and parents report feeling chronically stressed while juggling work and homelife. I believe it is this background of chronic, low‐level stress that may help to explain why our young people are struggling with their mental health.

Our stress response systems have evolved to deal with acute threats that come and go, not the kind of continuous, background worry that now characterizes many young people's lives (Sapolsky, 2003). When exposure is too intense or too prolonged, the evidence suggests it can lead to stress sensitization—making later stressors more, not less, likely to precipitate anxiety, depression or other difficulties—and to an accumulation of allostatic load, the wear and tear on the body and brain associated with chronically activated stress systems. At the same time, we have also steadily reduced children's everyday opportunities to face short‐lived challenges and discover ways that they can cope. Put another way, we are asking young people to manage a stress response system that was designed for intermittent crises in an environment that supplies almost constant, low‐level threat but offers fewer opportunities to practice coping with manageable stress. Is it any surprise that rates of anxiety and depression rise under these conditions?

Far fewer children now walk or cycle to school on their own. Time spent playing outside has fallen by roughly half in a generation, with less unstructured exploration of neighbourhoods and natural spaces and more time indoors (Smith, Sutherland, & Gill, 2021). Opportunities for risky physical play have been reduced by stricter safety standards and understandable parental concerns. Beyond physical risk‐taking, we have also curtailed other opportunities to learn from challenges. Research suggests that some well‐intentioned (and widely used) well‐being practices may inadvertently reinforce avoidance when they are not paired with supported exposure. In our schools for example, students may be excused from class presentations, reading aloud or completing homework if they report feeling anxious, rather than being helped to build up to these activities gradually. Schools increasingly provide safe spaces or withdrawal rooms and use trigger warnings to flag potentially upsetting material. Used carefully, these can be helpful, but there is mixed evidence that they reduce distress and come concern that they may signal that opting out is the safest response. My point here is not to criticize teachers, who are themselves working under increasingly intense pressure with often increasingly limited resources, but to highlight a system‐level drift towards avoiding everyday challenges that might otherwise offer children the opportunity to practise coping. Resilience research indicates that the children and young people who cope best with difficulties in life are often those who have had the opportunity to learn from smaller risks growing up—the so‐called ‘steeling effects’ or ‘stress inoculation’ described in experimental and longitudinal studies (Bowes & Jaffee, 2013; Rutter, 2012). The idea is not that adversity is good for children, but that encountering manageable, time‐limited stressors in a supportive context can strengthen their capacity to face later challenges without being overwhelmed. In medicine, we routinely weigh potential harms against potential benefits, and we accept that some short‐term discomfort or risk (an unpleasant procedure or a drug with side effects) may be justified by longer term gains. When it comes to children's everyday experiences, by contrast, we are far less willing to think in these terms. Faced with evidence of harm, our collective instinct is to ban an activity or programme altogether—to tighten the rules and take the most conservative option—rather than to ask whether, in a world of unavoidable and often chronic stress, the developmental benefits of participation and exposure might outweigh the risks if those experiences were better designed and more carefully supervised. If young people must navigate an increasingly demanding environment, then cultivating steeling effects and resilience becomes a necessity. The uncomfortable question this raises is: what forms of risk are we willing to tolerate, and under what conditions, if our aim is genuinely to help foster children's resilience?

If both chronic stress and risk avoidance can be seen as system‐level phenomena, we need to challenge the popular discourse that questions whether young people are ‘less resilient’ than previous generations. Resilience is both dynamic and relational; it emerges from interactions between individuals, families, schools, communities and broader structures. It is not simply a fixed trait within a child. Invoking ‘resilience’ in this way can become a way to reframe society's problems as a fault of the child, rather than a consequence of the social, educational and economic systems we have designed. I would argue that young people's distress and withdrawal from society are understandable responses to an environment that is both over‐protective in some ways and chronically threatening in others. We should be asking ourselves what it would mean to design school services and policies to mitigate some of the chronic stress while deliberately creating supported opportunities for manageable risk.

Perhaps, then, the task is not to ‘toughen up’ a supposedly fragile generation, but to redesign the systems that surround them. That means schools, communities and services that help to buffer children from chronic, unmanageable stress while building regular, supported encounters with ordinary challenges. This might include neighbourhoods where children are better able to walk or cycle short routes without adults, more spaces for unsupervised play and opportunities for adolescents to take on meaningful community responsibilities. It could include schools and universities that aim for all students, with good scaffolding, to speak in front of peers, take part in group projects or to go on trips, rather than routinely creating accommodations that may remove certain challenging tasks altogether. It requires funders and policymakers to think beyond short‐term safeguarding metrics to longer term developmental outcomes and to ask not only ‘Does this make it safer for today’ but also ‘What are the costs of never letting children take this risk?’ Most of all, it demands that we stop using the language of resilience to locate the problem in young people themselves. The world they are growing up in may well feel unprecedentedly chaotic. Their distress is not a resilience deficit, but a reasonable response. The real question for us is whether we are prepared to make our systems, rather than our children, do more of the adapting.

## Funding information

L.B. is supported by the NIHR Oxford Health Biomedical Research Centre (Mental Health in Development theme).

## Conflict of interest statement

The author has no conflicts of interest to declare.

## Ethics statement

No ethics approval was required for this debate article.

## Data Availability

Data sharing is not applicable to this article as no datasets were generated or analysed for this debate article.
